# Swine RNF5 positively regulates the antiviral activity of IFITM1 by mediating the degradation of ABHD16A

**DOI:** 10.1128/jvi.01277-24

**Published:** 2024-11-27

**Authors:** Xuemeng Shi, Lingyi Shen, Shuaiwu Chen, Mingyang Liu, Jingyi Wang, Xin Wen, Wei Liu, Lin Mao, Yunyun Ding, Li Yu, Jun Xu

**Affiliations:** 1 College of Life Science, Zhengdong New District Longzi Lake Campus, Henan Agricultural University 70573 , Zhengzhou, Henan, China; University of North Carolina at Chapel Hill, Chapel Hill, North Carolina, USA

**Keywords:** RNF5, ABHD16A, IFITM, post-translational modification, virus infection

## Abstract

Interferon-inducible transmembrane (IFITM) proteins are broad-spectrum antiviral factors that confer cellular resistance to virus invasion. α/β-Hydrolase domain-containing 16A (ABHD16A) has recently been identified as a novel depalmitoylase that can inhibit the antiviral activity of IFITM proteins by catalyzing the depalmitoyl reaction; this pattern may be crucial for the host to avoid damage caused by excessive immune response. However, it remains largely elusive about how host cells regulate the activity of ABHD16A. In the present study, we performed the AlphaFold2-based protein-protein interaction prediction and identified swine E3 ubiquitin ligase ring finger protein 5 (sRNF5) as a sABHD16A-interacting protein and negatively regulated the stability of sABHD16A. Using immunofluorescence and co-immunoprecipitation techniques, we uncovered that sRNF5 targeted sABHD16A for ubiquitination and degradation via the proteasomal pathway at residues K3 and K452. Furthermore, sABHD16A catalyzed the depalmitoylation of sIFITM1, which obstructed the antiviral function of sIFITM1, while sRNF5 caused ubiquitination of sABHD16A, which attenuated the depalmitoylation effect on sIFITM1, and consequently restored the antiviral activity of sIFITM1. Collectively, our findings demonstrate for the first time that sRNF5 positively regulates the antiviral function of sIFITM1 by mediating the degradation of sABHD16A, which expands the biological functions of RNF5 and ABHD16A in immune regulation. Moreover, our work highlights the well-designed interplay between RNF5, ABHD16A, and IFITM, which balances antiviral immune responses to avoid the disorders induced by excessive immune response.

**IMPORTANCE:**

Interferon and interferon-stimulated genes play significant and protective roles in the host’s defense against viral infection. IFITM family proteins, which can be strongly induced by interferon, have been identified as the first line of defense to prevent invasion of various viruses. Further analysis reveals the antiviral activity of IFITMs depends on palmitoylation/depalmitoylation. Recently, we reported that ABHD16A, as the first depalmitoylase of IFITMs, negatively regulated the antiviral activity of IFITMs. However, these raise crucial questions: how ABHD16A is regulated and remained in a balanced manner? Here, we show that swine RNF5 attenuates the negative regulation of sIFITM1 against virus invasion by modifying sABHD16A through ubiquitination and guiding sABHD16A for degradation. Thus, sRNF5-sABHD16A interplay plays an indispensable role in regulating immune response and avoiding the disorders induced by elevated interferon levels. Overall, our findings extend the upstream subtle regulatory molecular mechanism of IFITMs and provide potential targets for viral disease therapy.

## INTRODUCTION

To fight against viruses, the host initiates the production of interferons (IFN) to stimulate a large range of interferon-stimulated genes (ISGs) for confronting infection. Interferon-inducible transmembrane (IFITM) proteins are encoded by ISGs and can be rapidly induced by IFN to confer cellular resistance to various viruses infections in humans, swine, and other mammals (1, 2). Accumulating evidence has demonstrated that the plasma membrane as well as endolysosomal membrane-associated IFITM proteins restrict virus infection by interrupting fusion between the viral envelope and cellular membranes (3, 4). Notably, both we and others have reported that the membrane localization and antiviral activity of IFITM proteins rely on cysteine palmitoylation (S-palmitoylation), which increases hydrophobicity and targets IFITMs to lipid membranes (5–9). Protein S-palmitoylation refers to a type of post-translational modification in which one or more cysteine residues are covalently linked by the fatty acid palmitate (10). In cells, S-palmitoylation is dynamic and reversible, with palmitoyl acyltransferase (PAT) and depalmitoylase catalyzing palmitate linking and removing reactions, respectively (10). However, compared with the well-known PAT, our understanding of the depalmitoylase targeting IFITM proteins remains very limited.

Increasing evidence has revealed the members of α/β-hydrolase domain (ABHD)-containing family belong to depalmitoylases (11, 12). To determine whether members of the ABHD family specifically catalyze the depalmitoyl reaction of IFTIM proteins, we previously used sequence analysis to predict the serine metabolism enzyme ABHD16A, also known as human leucocyte antigen B (HLA-B)-associated transcript 5 (BAT5) (13), may catalyze the depalmitoylation reaction (14). Subsequently, we conducted co-immunoprecipitation (Co-IP) as well as Acyl-PEGyl exchange gel shift assays (APEGS) and identified ABHD16A as the first depalmitoylase targeting IFITM proteins in humans, swine, mouse, and other mammals (6). ABHD16A has been shown to interact and catalyze the depalmitoylation of IFITM proteins, thus disturbing IFITMs localized on the plasma membrane and consequently inhibiting the antiviral activity of IFITM proteins (6). Therefore, ABHD16A plays an indispensable negative role in regulating the antiviral effects of IFITM proteins, which provides a balanced role in the innate immune response to avoid disorders induced by elevated IFN levels. Nevertheless, the molecular mechanism underlying the regulation of ABHD16A during virus infection remains to be elucidated.

A previous study using a high-throughput yeast two-hybrid system has screened the interaction relationship of human ABHD16A, which suggested several proteins such as RING finger protein 5 (RNF5), scaffold attachment factor B (SAFB), and G protein-coupled receptor (GPRC5C) may interact with ABHD16A (15). The E3 ubiquitin ligase RNF5, also known as RMA1, has been implicated in endoplasmic reticulum (ER) stress response (16), autophagy (17), and innate immune responses via recognition and ubiquitination of targeting proteins (18, 19). Accumulating evidence has indicated that RNF5 targets several innate immune regulators, including MAVS, STING, and IRF3 for degradation, thus facilitating virus infection (19–21). However, a recent study on SARS-CoV-2 demonstrated that the envelope protein of virus was recognized and ubiquitinated by RNF5, which restricted virus replication (22). Therefore, RNF5 plays multiple roles in virus-host interaction, and it appears important to ascertain whether and how the interaction between ABHD16A and RNF5 influences IFITMs-mediated virus infection.

Japanese encephalitis virus (JEV) is an epidemic virus and can be transmitted among multiple species from which swine is the main source of infection and amplification host (23). JEV infection usually leads to abortion and stillbirth, along with mummified and weak fetuses in pregnant swine, thus causing serious effects on the breeding of swine and raising significant risks to public health and safety (23, 24). Previously, we demonstrated that swine IFITM1 (sIFITM1) specifically restricts JEV replication in swine PK15 cells, which depends on the S-palmitoylation modification (5). Subsequently, we discovered that sABHD16A negatively regulates sIFITM1 against JEV infection by catalyzing the depalmitoylation reaction (6). However, the factors and mechanisms responsible for regulating ABHD16A have yet to be elucidated.

The present work explores the mechanisms underlying the ubiquitination and S-palmitoylation post-translational modification of ABHD16A and IFITM1. We predicted and validated the interaction sites between RNF5 and ABHD16A among humans and swine by applying AlphaFold2 and Co-IP methods. Swine RNF5 targets sABHD16A at K3 and K452 residues for ubiquitination, thus delivering sABHD16A into proteasome for degradation. Furthermore, sRNF5 attenuated the depalmitoylase activity of sABHD16A, which elevated the S-palmitoylation level and the antiviral activities of sIFITM1. Our study reveals a novel mechanism of IFITM against virus invasion and suggests RNF5 as well as ABHD16A may act as promising therapeutic targets for the intervention of virus infection.

## RESULTS

### Identification of sRNF5 as a sABHD16A-interacting protein

**Fig 1:**
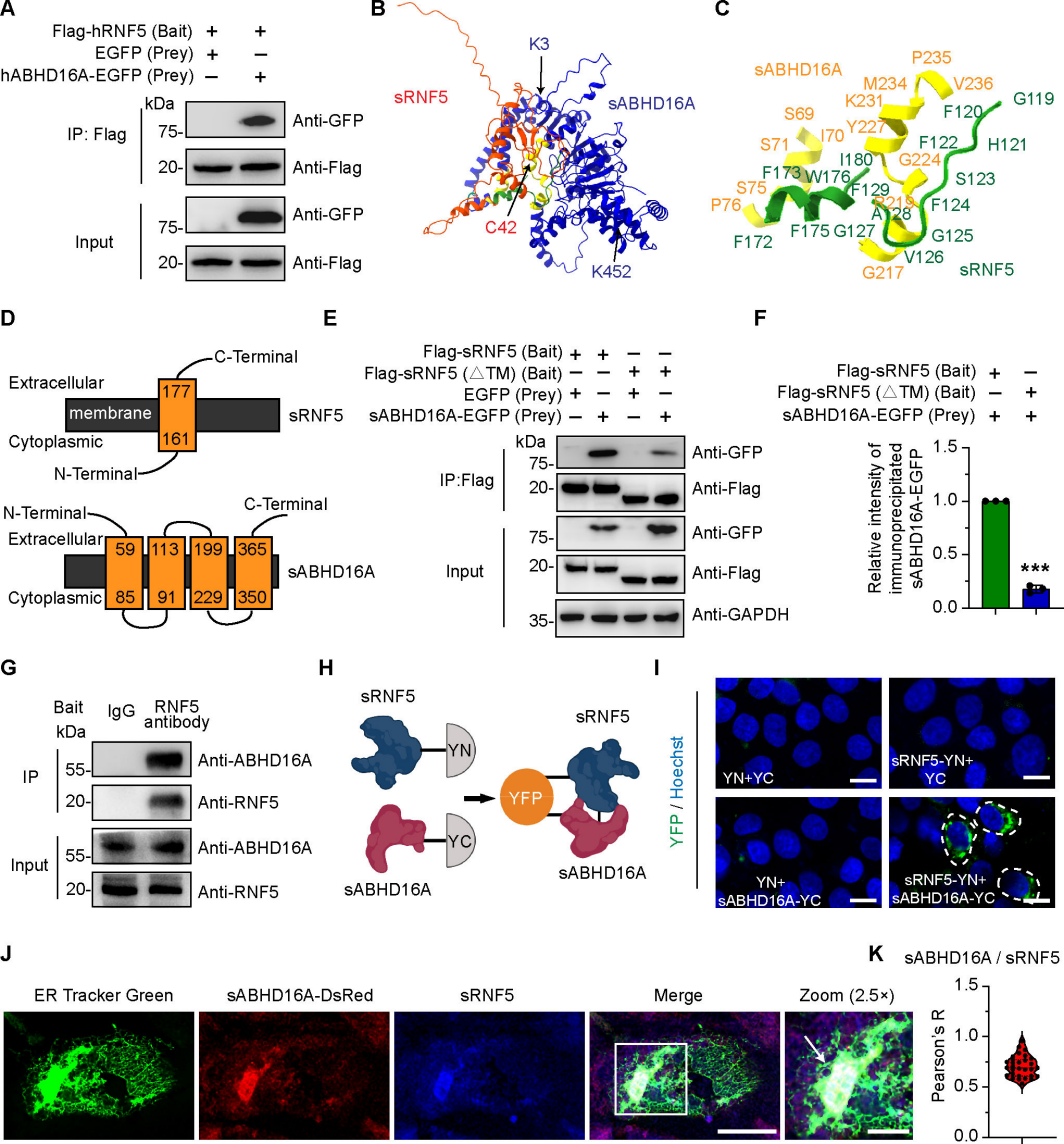
Identification of sRNF5 as a sABHD16A-interacting protein. (**A**) The interaction between human ABHD16A and RNF5 was confirmed by Co-IP assay in HEK293 cells. Expression constructs of Flag-hRNF5 with empty vector or hABHD16A-EGFP were transfected into HEK293 cells for 24 h, and cell lysates were immunoprecipitated through the Flag epitope followed by immunoblotting using anti-GFP and anti-Flag antibody. (**B**) The topologies of *Sus scrofa* RNF5 (Red) and ABHD16A (Blue) were predicted by AlphaFold2. (**C**) The interacting interface and residues between sRNF5 (green) with sABHD16A (yellow) were shown. (**D**) The transmembrane regions of *Sus scrofa* RNF5 and ABHD16A were predicted by Phyre2 using “normal mode.” (**E**) sRNF5 interacted with sABHD16A through its transmembrane domain. PK15 cells were cotransfected with indicated plasmids for 24 h, and cell lysates were immunoprecipitated (IP) with anti-Flag antibody. Expression of protein was analyzed by immunoblotting with Flag and green fluorescent protein (GFP) antibody. ΔTM, Δ161–177 aa of sRNF5. (**F**) The gray value of immunoprecipitated sABHD16A-EGFP bands to GAPDH internal reference band was normalized. Data are mean ± SD from three independent experiments. ***P < 0.001 (unpaired two-tailed *t* test). (**G**) The interaction between endogenous sRNF5 and sABHD16A was confirmed by Co-IP assay in PK15 cells; IgG and anti-RNF5 were used as negative control and bait antibody for IP, respectively. (**H**) The schematic diagram of bimolecular fluorescence complementation assay (BiFC) assay. (**I**) Detection of sRNF5 and sABHD16A interaction in living cells by using BiFC assay. PK15 cells were transiently transfected with indicated plasmids. Twenty-four hours later, cell nucleus were stained with Hoechst 33342 and subjected to confocal microscopy. yellow fluorescent protein (YFP) signals denote the interaction in living cells. Scale bar, 20 µm. YN, 1 to 155 aa of YFP; YC, 156 to 239 aa of YFP. (**J**) Representative confocal images show colocalization between sRNF5 and sABHD16A on ER. PK15 cells were transfected with sABHD16A-DsRed. Twenty-four hours after transfection, cells were stained with ER Tracker Green for 0.5 h, then washed and fixed with 4% paraformaldehyde, and subjected to immunofluorescence with RNF5 antibody. The insets are magnified views of the boxed areas. Scale bar, 20 µm (in cell images) and 10 µm (in the magnified box). (**K**) Pearson’s coefficient of sRNF5 and sABHD16A-DsRed. *n* = 30 cells were used for quantification.

A previous study has predicted that RNF5 may interact with ABHD16A by using a high-throughput yeast two-hybrid system (15). To validate the relationship between them, we carried out the Co-IP assay and detected that *Homo sapiens* ABHD16A (hABHD16A) bound to hRNF5 (Fig. 1A). Since the primary and secondary protein structures of RNF5 were highly conserved among *Homo sapiens* and *Sus scrofa*, we decided to investigate whether and how *Sus scrofa* RNF5 (sRNF5) physically interacts with sABHD16A. By using the emerging AlphaFold2, which has been used widely to predict protein structures, protein-protein interactions, and binding interfaces or sites (25, 26), we predicted the interface and residues utilized for sRNF5 and sABHD16A interaction (Fig. 1B and C). Owing to RNF5 and ABHD16A which have been reported to contain transmembrane domains and localize on ER (27, 28), we wondered whether the predicted contact residues were localized within the transmembrane domain. By using Phyre2 (29), we predicted the respective transmembrane regions of sRNF5 and sABHD16A (Fig. 1D) and subsequently discovered the binding sites were highly correlated with transmembrane regions. To further dissect the role transmembrane regions played in the relationship between sRNF5 and sABHD16A, we deleted the C-terminal transmembrane region (TM; 161–177 aa) of sRNF5 and subsequently performed Co-IP experiments. By using either anti-RNF5 or anti-Flag as the bait antibody, sABHD16A was readily co-immunoprecipitated by sRNF5; in contrast, depletion of the transmembrane region significantly attenuated the interaction (Fig. 1E through G). Collectively, these findings suggest that sRNF5 interacted with sABHD16A through its transmembrane domain.

Next, we investigated the subcellular localization of sRNF5 and sABHD16A in living cells. We generated the respective expression plasmids of sRNF5 and sABHD16A fused with YN (1–155 aa of YFP) and YC (156–239 aa of YFP) (Fig. 1H) and subsequently demonstrated the interaction between sRNF5 and sABHD16A in living PK15 cells by utilizing the bimolecular fluorescence complementation (BiFC) assay (Fig. 1I). In addition, by using immunofluorescence, we noticed that both sRNF5 and sABHD16A were localized on the ERs (Fig. 1J and K), which is consistent with previous reports that the two proteins are most distributed on the ER (28, 30). Taken together, our results indicate that the membrane-associated sRNF5 physically interacts with sABHD16A.

### The unidirectional regulation of sRNF5 to sABHD16A

**Fig 2:**
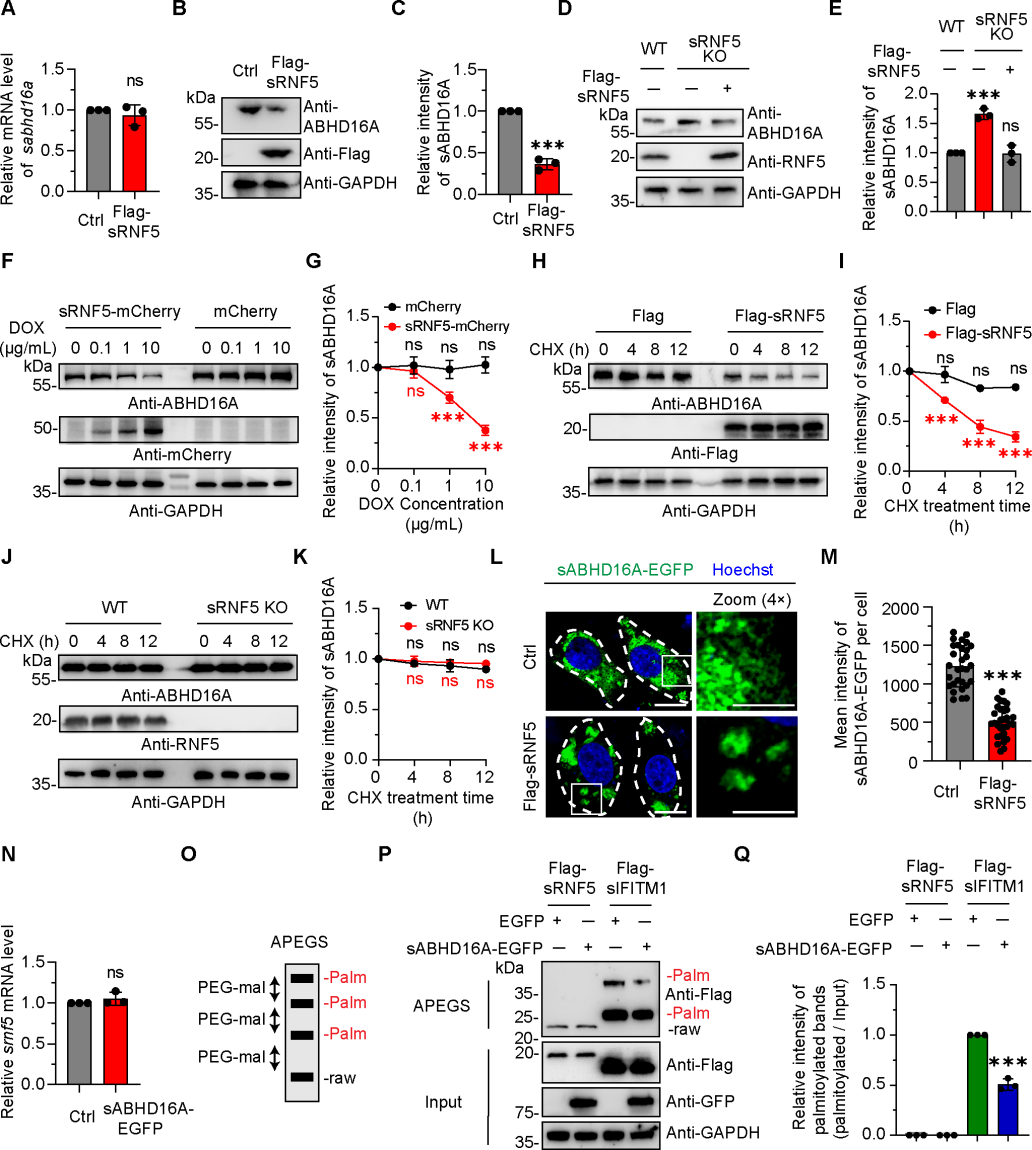
The unidirectional regulation of sRNF5 to sABHD16A. (**A**) Overexpression of sRNF5 showed no significant effects on the mRNA level of sABHD16A. (**B**) Endogenous sABHD16A was negatively regulated by the expression of sRNF5. The extracts of PK15 cells with Flag and Flag-sRNF5 expression were subjected to western blotting analysis, and ABHD16A antibody was used to detect the endogenous sABHD16A. (**C**) Quantification of the protein levels of sABHD16A; GAPDH antibody was used to verify equal sample loading. (**D and E**) Western blot analysis (**D**) and quantifications (**E**) of endogenous sABHD16A in WT, sRNF5 KO, and Flag-sRNF5 reintroduced cells. GAPDH was used as the internal control. (**F**) PK15 cells with or without sRNF5-mCherry expression were treated with the indicated concentration of Dox and harvested after 24 h. (**G**) The relative protein expression level of endogenous sABHD16A was analyzed by western blotting and presented in the degradation curve. (**H**) Flag or Flag-sRNF5 expression plasmid was introduced into PK15 cells for 24 h and then treated with the indicated concentration of Cycloheximide (CHX) and harvested. (**I**) The relative protein expression level of endogenous sABHD16A was analyzed by western blotting and presented in the degradation curve. (**J**) Wild-type and sRNF5 KO PK15 cells were treated with 50 µg/mL CHX and harvested at different time points. (**K**) The relative protein expression level of endogenous sABHD16A was analyzed by western blotting and presented in the degradation curve. GAPDH was used as internal control, and the initial levels of endogenous sABHD16A of each condition are normalized to 1. (**L**) Representative confocal micrographs showing expression of sRNF5 results in aggregated distribution of sABHD16A. Insets show the magnified view of the boxed areas. White dashed lines indicated the cell outline. Scale bar, 20 µm (in cell images) and 10 µm (in the magnified box). (**M**) Quantification of the mean intensity of sABHD16A-EGFP. *n* = 30 cells were used for quantification. (**N**) qRT-PCR analysis showed no change in the amount of sRNF5 mRNA upon overexpression of sABHD16A. (**O**) Schematic representation of APEGS method. The S-palmitoylated cysteine sites were replaced by 5 kDa polyethylene glycol maleimide (PEG-mal), and the S-palmitoylation was detected by western blotting. (**P**) sRNF5 was not S-palmitoylated, and its protein level was not affected by overexpression of sABHD16A. PK15 cells were cotransfected with indicated expression plasmids, and 24 h later, cells were harvested and lysed. Cell lysates were subjected to APEGS process and analyzed by western blotting. The gray value of all palmitoylated bands to input bands was considered as the palmitoylated level. Statistical analysis data are mean ± SD from three independent experiments. ns, not significant; ***P < 0.001 (one-way analysis of variance (ANOVA) for E, G, I, and K; unpaired two-tailed *t* test for A, C, M, N, and Q).

The E3 ubiquitin ligase RNF5 has been demonstrated to recognize target proteins and promote their degradation (31). To determine the effect of sRNF5 on sABHD16A, we carried out quantitative real-time PCR (qRT-PCR) as well as western blotting experiments and showed that the endogenous protein level rather than mRNA amounts of sABHD16A was significantly reduced upon sRNF5 overexpression (Fig. 2A through C). To further reveal the regulatory effect of sRNF5 on sABHD16A, we generated sRNF5 knockout PK15 cells by using CRISPR/Cas9 and found the protein level of endogenous sABHD16A increased by ∼1.6-fold in sRNF5 knockout cells when compared with wild-type (WT) (Fig. 2D and E). In addition, the amounts of endogenous sABHD16A were restored when Flag-sRNF5 was reintroduced into sRNF5 KO cells (Fig. 2D and E). These results suggested that sRNF5 negatively regulates sABHD16A.

Next, we carried out the Tet-on-inducible experiment to track the regulation of sRNF5 to sABHD16A. The cDNA sequence of swine RNF5 was cloned into pTight-mCherry-EF1-tetR-2A vector and then transfected into PK15 cells. Twenty-four hours later, doxycycline hyclate (Dox) was added to induce sRNF5-mCherry expression. The protein level of sRNF5-mCherry was significantly increased after 24 h treatment with gradual doses of Dox (0, 0.1, 1, and 10 µg/mL) (Fig. 2F). Meanwhile, we assessed the endogenous protein level of sABHD16A and found with the expression of sRNF5, the level of sABHD16A gradually decreased (Fig. 2F and G). Taken together, by using the Tet-on system, we tracked the protein level of endogenous sABHD16A followed by the induction of sRNF5 and speculated that sRNF5 may promote sABHD16A degradation.

To verify whether sRNF5 influences the decay rate of endogenous sABHD16A, we conducted the protein stability assay with Cycloheximide (CHX) treatment and measured that the degradation rate of endogenous sABHD16A was significantly faster in sRNF5-overexpressing cells than in the control group (Fig. 2H1). However, knockout of RNF5 did not further slow down the degradation rate of sABHD16A (Fig. 2J and K), which may be due to the fact that the sABHD16A degradation rate is already very slow under normal circumstances.

Recently, Nguyen et al. demonstrated that ABHD16A localized on the ER membrane and was responsible for regulating mitochondrial dynamics (28). A similar morphology and distribution of sABHD16A were observed in PK15 cells, whereas the localization of sABHD16A markedly rearranged to form aggregated structures and the fluorescence intensity significantly decreased in sRNF5-overexpressing cells (Fig. 2L and M). Altogether, these results suggested that sRNF5 negatively regulates the stable state and distribution of sABHD16A.

To further explore whether sABHD16A exerted any effects on sRNF5, the mRNA transcription level of sRNF5 was first quantified, and there is no apparent change in the case of sABHD16A-overexpressing cells (Fig. 2N). Apart from the well-known acylglycerol lipase and phosphatidylserine lipase activities, we recently identified the depalmitoylation function of ABHD16A, which catalyzed the depalmitoylation of sIFITM1 (Fig. 2P and Q) (6). Thus, we subsequently aimed to determine whether sABHD16A affects the total protein and palmitoylation modification levels of sRNF5. By implementing the Acyl-PEGyl exchange gel shift (APEGS) assays, a novel method in which S-palmitoylated proteins were replaced with methoxy PEG-mal (Fig. 2O) (32), we witnessed sRNF5 was not S-palmitoylated in either control or sABHD16A-overexpressing cells. In addition, the total amount of sRNF5 protein was not regulated by sABHD16A (Fig. 2P and Q). Together, the above data revealed an unidirectional regulation of sRNF5 to sABHD16A. sRNF5 targets sABHD16A for ubiquitination and degradation.

**Fig 3:**
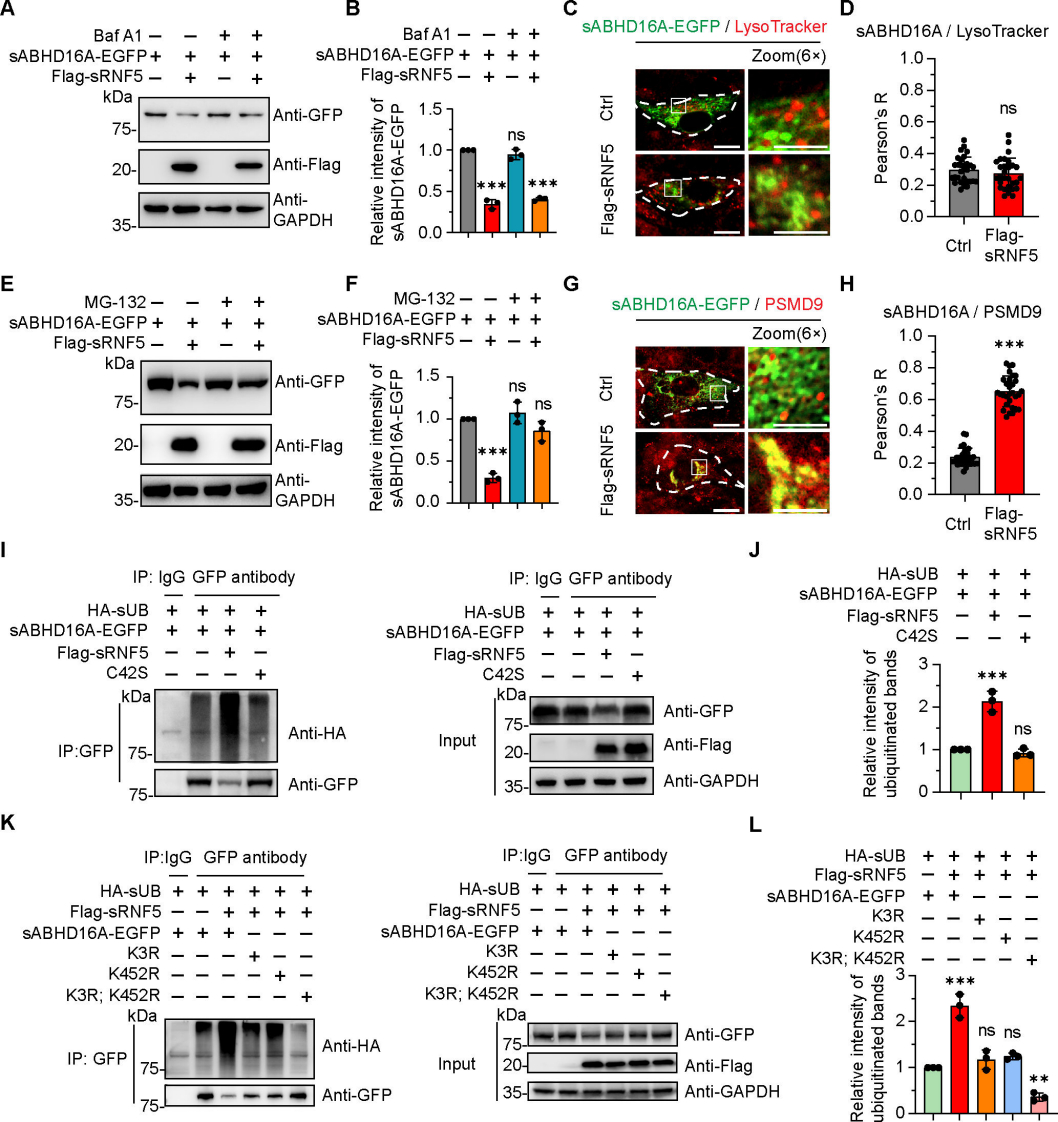
sRNF5 targets sABHD16A for ubiquitination and degradation. (**A**) Blocking the lysosome degradation pathway by treatment with BafA1 did not affect the protein level of sABHD16A. PK15 cells cotransfected with indicated plasmids were treated with 100 nM BafA1 for 12 h. (**B**) Quantification of the protein levels of sABHD16A-EGFP in each condition; GAPDH antibody was used to verify equal sample loading. (**C**) Confocal images showing the subcellular distribution of sABHD16A-EGFP with LysoTracker Red in the indicated context. Transfected cells were treated with 50 nM LysoTracker Red for 1 h before image was taken. (**D**) Quantification of Pearson’s correlation coefficients to determine the level of colocalization for sABHD16A-EGFP with LysoTracker Red signals as depicted in C. *n* = 30 cells. (**E**) Blocking the proteasome degradation pathway by MG-132 treatment restored the protein level of sABHD16A. PK15 cells cotransfected with indicated plasmids were treated with 10 µM MG-132 for 12 h. (**F**) The protein levels of sABHD16A-EGFP in each group were analyzed; GAPDH antibody was used to verify equal sample loading. (**G**) Overexpression of sRNF5 leads to sABHD16A delivered into PSMD9-marked proteasome. (**H**) Pearson’s coefficient of sABHD16A-EGFP and PSMD9. *n* = 30 cells. (**I**) Overexpression of full-length sRNF5, but not sRNF5 (C42S), elevated the ubiquitination level of sABHD16A. PK15 cells were transfected with HA-Ub, sABHD16A-EGFP, and Flag-sRNF5 or sRNF5 (C42S) for 24 h. Cell lysates were subjected to GFP immunoprecipitation and analyzed via western blotting. IgG was used as the negative control. (**J**) The gray values of all ubiquitinated bands to GAPDH bands were considered as the ubiquitinated level. (**K**) K3 and K452 were the main ubiquitin sites of sABHD16A. PK15 cells were transfected with indicated plasmids for 24 h. Cell lysates were immunoprecipitated with anti-GFP. The immunoprecipitants were analyzed by immunoblotting with anti-HA. IgG was used as the negative control. (**L**) The gray values of all ubiquitinated bands to GAPDH bands were considered as the ubiquitinated level. In C and G, insets show the magnified view of the boxed areas. White dashed lines indicated the cell outline. Scale bar, 20 µm (in cell images) and 10 µm (in the magnified box). Statistical analysis data are mean ± SD from three independent experiments. ns, not significant; **P < 0.01 and ***P < 0.001 (one-way ANOVA for B, F, J, and L; unpaired two-tailed *t* test for D and H).

The evidence for decreased amount of sABHD16A in sRNF5-expressing cells prompted us to hypothesize that sABHD16A was misdirected toward the degradation pathway. To further confirm whether overexpression of sRNF5 elevated the degradation of sABHD16A via the lysosomal or proteasomal pathway, transfected PK15 cells were respectively treated with 100 nM Bafilomycin A1 (Baf A1) and 10 µM MG-132 for 12 h to inhibit the degradation activity of lysosome and proteasome (33, 34), and then, cell lysates were harvested for immunoblotting. It is noted that the protein level of sABHD16A was significantly restored in MG-132 rather than in Baf A1-treated sRNF5-overexpressing cells (Fig. 3A, B, E and F), suggesting sABHD16A may be degraded in proteasome upon sRNF5 expression. To verify this hypothesis, we further designed imaging and immunostaining assays with respective LysoTracker and PSMD9 as probes for lysosome and proteasome (35, 36). The result showed no apparent colocalization between sABHD16A with LysoTracker-marked lysosome and PSMD9-labeled proteasome in wild-type PK15 cells (Fig. 3C and G), indicating that most of the sABHD16A was not sorted for degradation. In contrast, the aggregated sABHD16A patches were largely overlapped with PSMD9-positive proteasomes instead of lysosomes in sRNF5-overexpressing cells (Fig. 3C, D, G and H). Altogether, these observations provided compelling evidence that overexpression of sRNF5 results in sABHD16A being misdirected toward the proteasome for degradation.

RNF5 belongs to the E3 ubiquitin ligase family, and the critical C42 residue localized in the RING domain was responsible for the enzymatic activity of RNF5 (18, 37). We speculated that sRNF5 may target sABHD16A to promote its proteasomal pathway degradation via ubiquitination. Overexpression of full-length rather than the C42S mutant sRNF5 significantly elevated sABHD16A’s ubiquitination and reduced the total amount of sABHD16A (Fig. 3I and J), denoting sRNF5 indeed acts as an E3 ligase to target sABHD16A for ubiquitination.

To further clarify the ubiquitin site of sABHD16A and confirm whether this site is located in the binding region responsible for interaction, the GPS-Uber website was utilized (38). According to the scores for all 16 lysine residues of *Sus scrofa* ABHD16A, K3 and K452 were selected for subsequent investigation (K3 scored 0.5296; K452 scored 0.6872). We mutated these two lysines into arginines individually and introduced the corresponding expression plasmids into PK15 cells. Transfected cells were lysed and immunoprecipitated with anti-GFP followed by immunoblotting with anti-HA. The results indicated that either K3R or K452R mutation restored the ubiquitin modification of sABHD16A in sRNF5 overexpression cells (Fig. 3K and L). Furthermore, expression of K3R; K452R double mutation led to sABHD16A can’t be ubiquitinated by sRNF5 (Fig. 3K and L). Consistently, sRNF5-mediated degradation of sABHD16A was abolished when K3 and K452 were mutated to arginine (Fig. 3K and L), thus implying that sRNF5 promotes sABHD16A degradation via ubiquitination and the contact region utilized for sRNF5-sABHD16A interplay is not sufficient for ubiquitination modification.

### sRNF5 increases the S-palmitoylation level of sIFITM1 by negatively regulating sABHD16A

**Fig 4:**
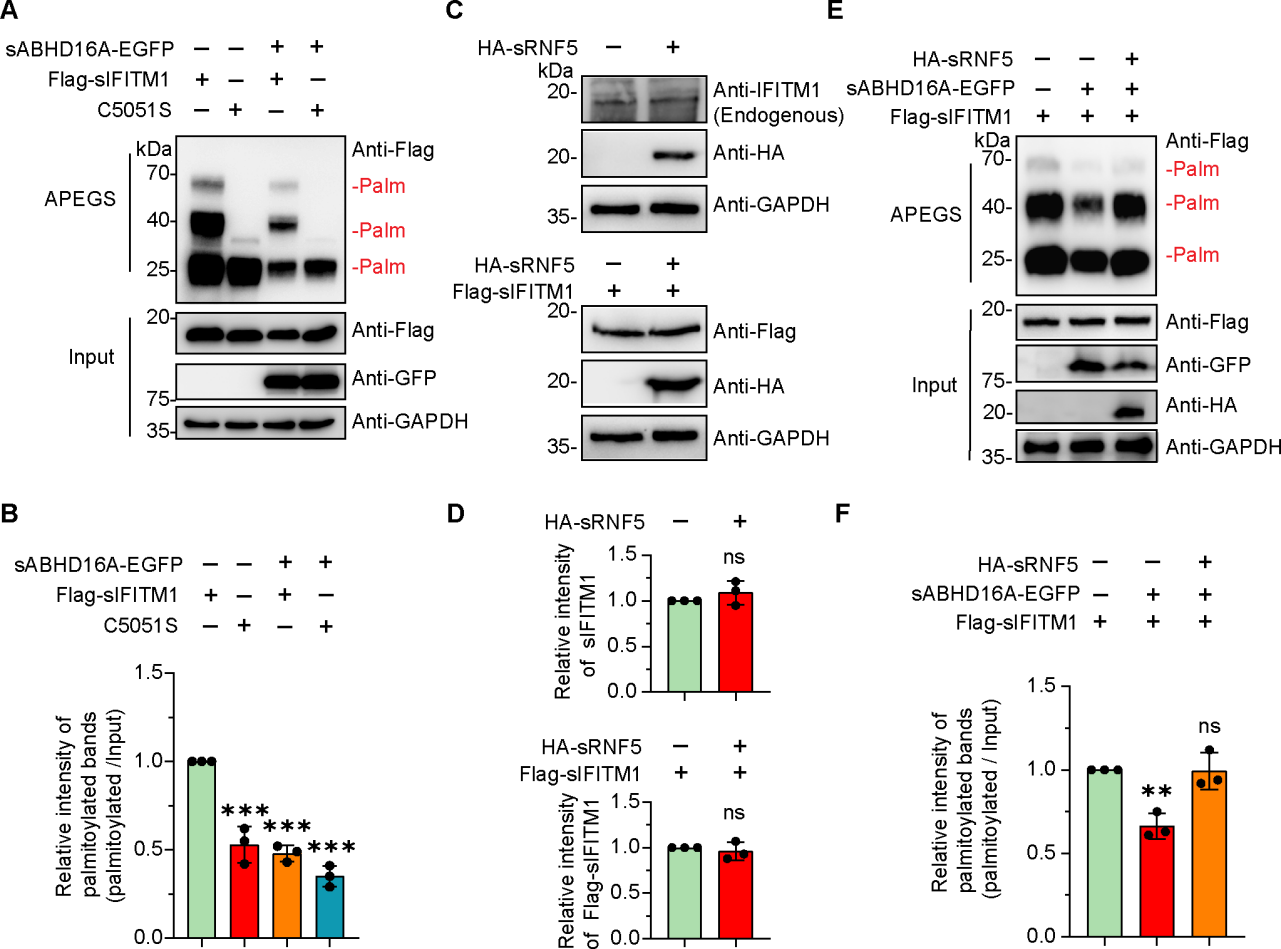
sRNF5 increases the S-palmitoylation level of sIFITM1 by negatively regulating sABHD16A. (**A**) sABHD16A catalyzed the depalmitoylation of sIFITM1. PK15 cells were transfected with indicated plasmids for 24 h; cells were harvested and subjected to APEGS assay. (**B**) The gray values of all palmitoylated sIFITM1 bands to input sIFITM1 bands were considered as the palmitoylated level. (**C**) Overexpression of sRNF5 had no effect on the expression of either endogenous or exogenous sIFITM1. (**D**) The gray value of sIFITM1 band to GAPDH internal reference band was normalized. (**E**) sRNF5 restored the palmitoyl modification level of sIFITM1 by negatively regulating sABHD16A. PK15 cells were cotransfected with indicated expression plasmids, and 24 h later, cells were harvested and lysed. Cell lysates were subjected to APEGS process and analyzed by western blotting. (**F**) The gray values of all palmitoylated sIFITM1 bands to input sIFITM1 bands were considered as the palmitoylated level. Statistical analysis data are mean ± SD from three independent experiments. ns, not significant; **P < 0.01 and ***P < 0.001 (one-way ANOVA for B and F; unpaired two-tailed *t* test for D).

Given that sRNF5 targets sABHD16A for ubiquitination and degradation, we next investigated whether sRNF5 influences the depalmitoylase activity of sABHD16A. IFITM proteins are known to be modified by S-palmitoylation (7), and the distribution and the antiviral function of IFITM proteins depend on S-palmitoylation modification (8, 39). Since we previously demonstrated that sIFITM1 played a more prominent role in restricting virus invasion when compared with sIFITM2 and sIFITM3 (5), sIFITM1 was focused in the following investigations. The conserved cysteine residues (e.g., C50 and C51) were known to be responsible for the S-palmitoylation of sIFITM1 (5); by using the APEGS system, we revealed that sABHD16A catalyzed the depalmitoylation of sIFITM1 instead of mutant sIFITM1 (C5051S) (Fig. 4A and B). To rule out the possibility that sRNF5 may directly regulate sIFITM1, we examined the protein levels of both endogenous and exogenous sIFITM1 in sRNF5-expressing PK15 cells and found that sRNF5 did not affect the stability of sIFITM1 (Fig. 4C and D). To determine whether sRNF5 regulates sABHD16A-mediated depalmitoylation of sIFITM1, we performed the APEGS assay and demonstrated that overexpression of sRNF5 significantly restored the PEG-mal-linking bands of sIFITM1 in sABHD16A-expressing cells (Fig. 4E and F), thus indicating that sRNF5 attenuated the depalmitoylase activity of sABHD16A, which elevated the S-palmitoylation of sIFITM1.

### The downregulation of sABHD16A mediated by sRNF5 is critical for the activity of sIFITM1 to restrict virus infection

**Fig 5:**
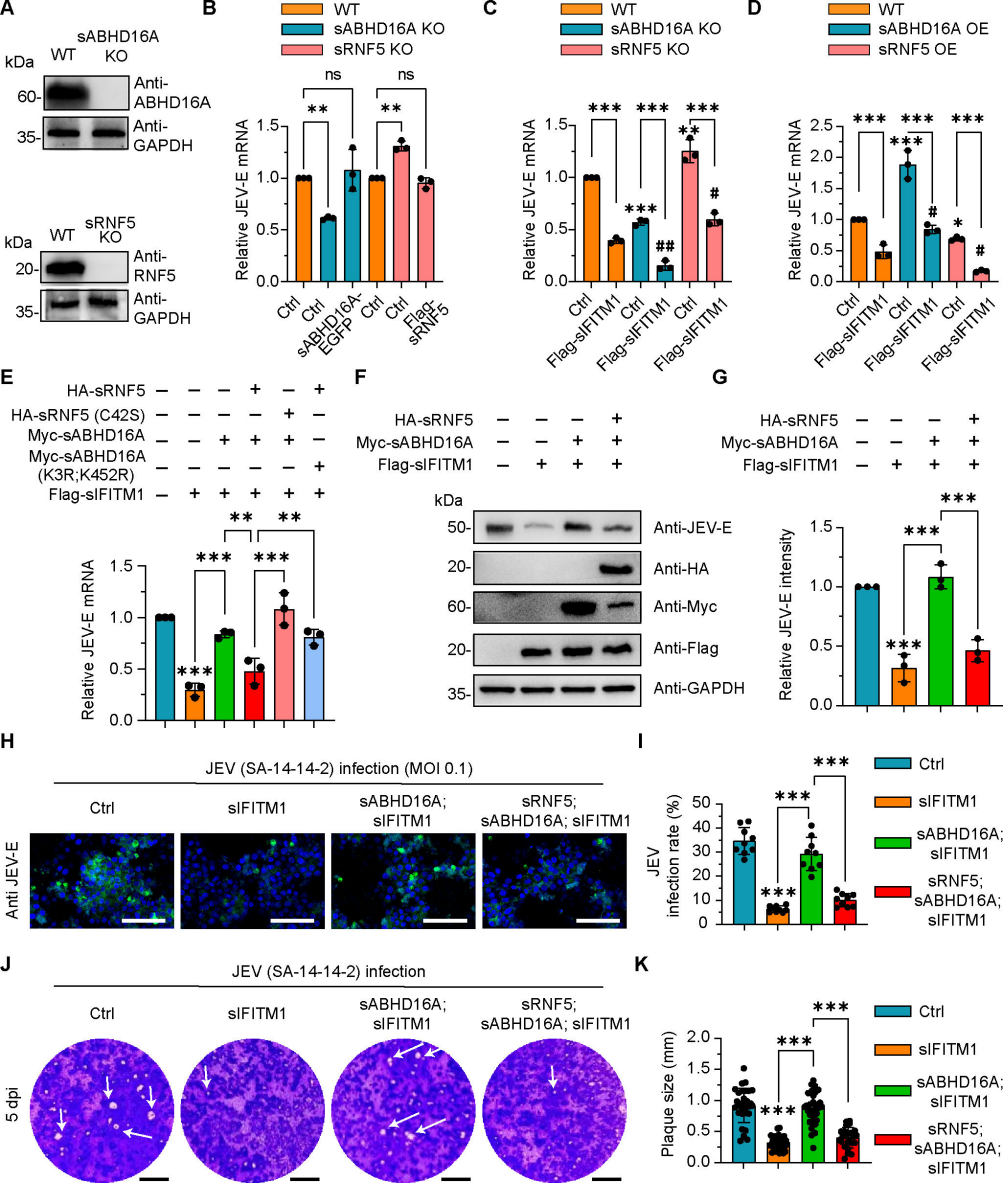
The downregulation of sABHD16A mediated by sRNF5 is critical for the activity of sIFITM1 to restrict JEV infection. (**A**) WT, sABHD16A KO, and sRNF5 KO PK15 cells were lysed, and the protein levels of sABHD16A or sRNF5 were analyzed through western blotting. GAPDH antibody was used to verify equal sample loading. (**B–E**) PK15 cells were transfected with indicated plasmids for 24 h, and the intracellular JEV-E mRNA in each group was measured by qRT-PCR 24 h after infection. MOI = 0.1. (**F**) The intracellular JEV-E protein level in each condition of transfected PK15 cells was measured by western blotting; 24 hour post-infection (hpi), MOI = 0.1. (**G**) The relative intensity of JEV-E in the indicated condition was analyzed via ImageJ. The gray value of JEV-E band to GAPDH internal reference band was normalized. (**H**) Transfected PK15 cells were infected with JEV live vaccine for 24 h. Cells were fixed, immunostained with JEV-E glycoprotein antibody, and subjected to confocal microscopy. Scale bar, 100 µm. (**I**) Quantification of the JEV infection rate for the indicated group. (**J**) PK15 cells were transfected with indicated plasmids for 24 h, and cell monolayers were infected with JEV live vaccine for 1 h, and cells were washed and immersed in complete Dulbecco's modified Eagle medium (DMEM) containing 2% carboxymethyl cellulose sodium salt and 2% fetal bovine serum (FBS). After 5 days, cells were stained with crystal violet. (**K**) The plaque morphology (arrows) was analyzed by using ImageJ. Three independent regions were selected randomly from three wells for each group. Statistical analysis data are mean ± SD from three independent experiments. ns, not significant; *P < 0.05, **P < 0.01 and ***P < 0.001 (wild-type cells as the Ctrl; one-way ANOVA). ^#^*P* < 0.05 and ^##^*P* < 0.01 (single transfection of Flag-sIFITM1 as the Ctrl, one-way ANOVA).

Previously, we have identified the S-palmitoylation modification of sIFITM1 plays an indispensable role in restricting JEV infection in PK15 cells (5). Afterwards, we revealed that sABHD16A catalyzed the depalmitoyl reaction, which inhibited the anti-JEV capabilities of sIFITM1 (6). Does sRNF5-sABHD16A interplay influence the antiviral function of sIFITM1? To address this, we knocked out sABHD16A and sRNF5 in PK15 cells by using the CRISPR/Cas9 way (Fig. 5A). Cells were infected by JEV (multiplicity of infection [MOI] 0.1) for 24 h and subsequently were lysed for qRT-PCR. It is apparent that the viral mRNA level decreased in sABHD16A deficiency cells when compared with the wild type. In contrast, the expression of sABHD16A-EGFP in sABHD16A KO cells restored the phenotype (Fig. 5B). We further investigated the impact of sABHD16A on the antiviral effect of sIFITM1. By checking intracellular JEV-E mRNA levels, we witnessed that loss of sABHD16A promotes sIFITM1 inhibition of virus infection (Fig. 5C), indicating that sABHD16A negatively regulates the antiviral activity of sIFITM1, which is consistent with the previous conclusion obtained on HEK293 cells (6). We also carried out the JEV infection assay on wild-type and sRNF5 knockout cells. The intracellular JEV mRNA amount significantly increased in sRNF5 deficiency cells compared with that in wild-type cells (Fig. 5B). In addition, transfection of sRNF5 expression plasmids into sRNF5 KO cells reduced the viral mRNA level, which is comparable to that in wild-type cells (Fig. 5B). Although sIFITM1 expression restricted virus infection in both WT and sRNF5 KO cells, more JEV mRNA copies were detected in sRNF5 deficiency cells (Fig. 5C), suggesting sRNF5 positively regulates the antiviral function of sIFITM1.

Next, we examined the effect of overexpression of sABHD16A and sRNF5 on the antiviral activity of sIFITM1. PK15 cells were single or double transfected with expression plasmid of HA-sRNF5, Myc-sABHD16A, and Flag-sIFITM1 for 24 h and then infected by JEV vaccine for 24 h. Intracellular mRNA was extracted, reverse transcribed, and analyzed by qRT-PCR. Overexpression of sABHD16A counteracted the antiviral activity of sIFITM1, which has been demonstrated in our previous report (6) (Fig. 5D). In contrast, the amounts of JEV-E mRNA were partially restricted upon sRNF5 expression. Furthermore, expression of sRNF5 significantly promotes sIFITM1 fighting against virus infection (Fig. 5D). Taken together, our results revealed that sRNF5 positively regulates the antiviral function of sIFITM1.

To further verify whether the negative regulation of sABHD16A mediated by sRNF5 influences sIFITM1 against virus infection, PK15 cells were transfected with respective plasmids and 24 h later infected with JEV live vaccine strain SA-14-14-2 (MOI 0.1) for 24 h. Then, we individually estimated the transcription of gene encoding JEV envelope protein (JEV-E) via qRT-PCR and the protein amount of JEV-E by immunoblotting in cell lysis and found that the reduced anti-JEV capabilities of sIFITM1 caused by sABHD16A expression was almost restored upon overexpression of sRNF5 (Fig. 5E through G). In contrast, overexpression of either the sRNF5 (C42S) or sABHD16A (K3R; K452R) counteracted the antiviral activity of sIFITM1 (Fig. 5E), which may be due to the persistent depalmitoylation of sIFITM1 by sABHD16A that is not affected by sRNF5-mediated ubiquitination. Moreover, by respectively implementing the immunostaining with JEV-E antibody and plaque formation, we detected that the proportion of JEV-E-positive cells and plaque sizes in the sIFITM1-expressing group displayed significantly reduced levels compared with those of the control, demonstrating the anti-JEV ability of sIFITM1. Overexpression of sABHD16A counteracted the antiviral function of sIFITM1. Nevertheless, the introduction of sRNF5 apparently recovered the antiviral activity of sIFITM1 in the context of sABHD16A overexpression (Fig. 5H through K).

**Fig 6:**
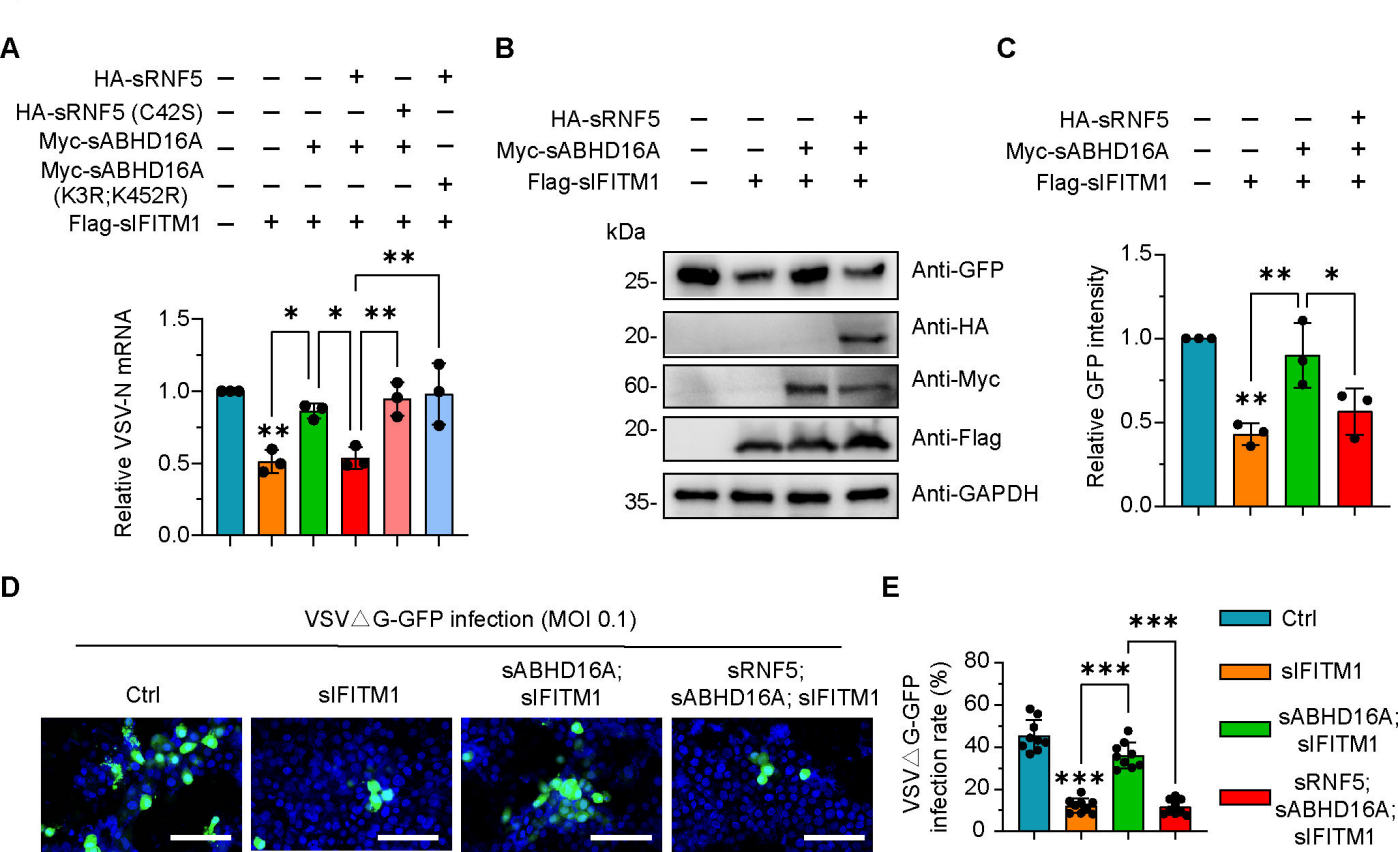
The downregulation of sABHD16A mediated by sRNF5 is critical for the activity of sIFITM1 to restrict VSVΔG-GFP pseudotyped virus infection. (**A**) PK15 cells were transfected with indicated plasmids for 24 h, and the intracellular VSV-N mRNA in each group was measured by qRT-PCR 24 h after infection. MOI = 0.1. (**B**) The intracellular GFP protein level in each condition of transfected PK15 cells was measured by western blotting; 24 hpi, MOI = 0.1. (**C**) The relative intensity of GFP in the indicated condition was analyzed via ImageJ. The gray value of GFP band to GAPDH internal reference band was calculated. (**D**) Transfected PK15 cells were infected with VSVΔG-GFP pseudotypes for 24 h. Cells were fixed, stained with Hoechst, and subjected to confocal microscopy. Scale bar, 100 µm. (**E**) Quantification of the VSVΔG-GFP pseudotype infection rate for the indicated group. Three independent regions were selected randomly from three wells for each group. In A, C, and E, data are mean ± SD from three independent experiments. *P < 0.05, **P < 0.01, and ***P < 0.001 (one-way ANOVA).

Apart from JEV, we have adopted another representative RNA virus, vesicular stomatitis virus (VSV), to investigate the role of sRNF5-sABHD16A in regulating sIFITM1 against virus infection, since the budding of VSV virions from cell surface has been shown not to require glycoprotein, thus leading to the development of recombinant viruses in which the VSV glycoprotein gene was replaced with genes encoding GFP (40, 41). By individually analyzing and calculating the VSVΔG nucleocapsid mRNA amount, GFP protein level, and GFP-positive cell proportion, we demonstrated that sABHD16A overexpression counteracted the antiviral activity of sIFITM1; however, the expression of sRNF5 rather than enzyme-catalyzed mutant sRNF5(C42S) could relieve this inhibitory effect (Fig. 6). Collectively, these data revealed that swine RNF5 positively regulates the antiviral activity of sIFITM1 by mediating the degradation of sABHD16A.

## DISCUSSION

Innate immunity is the first line of host defense against invading pathogens and is designed to maintain homeostasis by precisely regulating key molecules. A variety of protein post-translational modifications are involved in the stress response of host cells to viruses, including phosphorylation, acylation, and ubiquitination (42). Accumulating evidence has indicated that IFITM proteins combating virus invasion depend on S-palmitoylation modification. S-Palmitoylation of protein is reversible, and this dynamic regulation is essential for immune balance (10). However, molecular mechanisms underlying the regulation of depalmitoylation reaction on IFITMs remain poorly understood. Here, we demonstrate in swine cells that E3 ubiquitin ligase sRNF5 targets sABHD16A, a novel found depalmitoylase of sIFITM1, for degradation. Moreover, we demonstrate for the first time that sRNF5 relieves sIFITM1 from sABHD16A-catalyzed depalmitoylation reaction, which enhances the plasma membrane distribution and antiviral activities of sIFITM1.

Taking advantage of bioinformatics methods, we predicted the contact interface between swine RNF5 and ABHD16A, which was highly correlated with their respective transmembrane regions. These results suggested that sRNF5 interacts with sABHD16A via their respective transmembrane domains, which is consistent with the previously reported both RNF5 and ABHD16A localized on the ER membrane (28, 30). Further studies will be necessary to confirm the accuracy of prediction by using crystal structure and domain deletion ways.

The coding sequence of human ABHD16A is closely associated with tumor necrosis factor and involved in the major histocompatibility complex class III region (43, 44), which indicates ABHD16A may take part in the immune regulation process. In 1995, a study on mice indicated the expression of ABHD16A could influence the immunogenicity of mouse marrow cells (45), which for the first time revealed the role of ABHD16A in immune regulation. Furthermore, Turcotte et al. denoted ABHD16A as an immune-balancing regulator in neutrophils by catalyzing the hydrolysis of PG-glycerol (46). In 2015, Kamat et al. demonstrated that ABHD16A exhibits lysophospholipase activity, which dynamically regulates the generation of immunomodulatory lysophosphatidylserines (Lyso-PS) (47). Notably, our previous study on swine PK15 cells confirmed that treatment with an ABHD16A inhibitor attenuated the generation of Lyso-PS, which influenced the replication of JEV (6). Collectively, these data suggested the stable state of ABHD16A is essential for immune regulation. In the present study, we separately inhibited protein synthesis and degradation pathways and found the protein level of swine ABHD16A was not significantly altered. In addition, sABHD16A did not colocalize with either lysosome or proteasome marker in wild-type cells, indicating sABHD16A remains in a relatively stable state without being delivered into lysosome or proteasome for degradation.

Increasing efforts have been made to determine the effects of ABHD16A on immunoregulation as well as disease occurrence and development (14); however, the cellular machinery (i.e., post-translational modification) that controls the activity of ABHD16A remains unknown at this time. Here, we discovered swine ABHD16A interplays with E3 ubiquitin ligase sRNF5 and sRNF5 targets sABHD16A for ubiquitination via the proteasomal degradation pathway. Moreover, by using GPS-Uber, we predicted and validated two sites (K3 and K452) that were ubiquitinated by sRNF5 within sABHD16A. Interestingly, these two sites were not located in the region of contact interface (69–77 and 215–236 aa of sABHD16A), indicating the interaction with sRNF5 is not sufficient to trigger the ubiquitination and degradation of sABHD16A.

Although ABHD16A has been reported to play roles in the immune regulation for decades (14), whether ABHD16A mediates viral infection has not yet been answered for a long time. Our recent research for the first time showed that ABHD16A negatively regulates IFITM proteins against RNA virus infections such as JEV via depalmitoylation of IFITMs in both humans and swine (6). Herein, we further identified sRNF5 that can act upstream of sABHD16A-sIFITM1 and target sABHD16A for ubiquitination and degradation, thus weakening the depalmitoylation effect of sABHD16A on sIFITM1 and consequently restoring the localization and anti-JEV activities of sIFITM1.

Accumulating evidence now indicates that RNF5 ubiquitinates and degrades key regulators in the innate immune, including MAVS, STING, and IRF3, either by itself or being hijacked by viral proteins, which inhibits the induction of IFN and facilitates the replication of various viruses (19, 21, 48, 49). IFITMs are well-known factors involved in the innate immune system and are directly induced by IFN (4); however, to date, whether RNF5 regulates the antiviral function of IFITM proteins has yet to be determined. In the present study, we first evaluated the effects of overexpression of swine RNF5 on sIFITM1 and found both the endogenous and exogenous sIFITM1’s protein amounts were not altered in response to sRNF5 overexpression, suggesting sRNF5 did not influence the induction of sIFITM1. Furthermore, overexpression of sRNF5 elevates the S-palmitoylation of sIFITM1 by delivering sABHD16A into the degradation pathway, which promotes the activity of sIFITM1 against virus invasion. Therefore, we discovered a novel positive role played by RNF5 in the innate immune response.

In conclusion, our study demonstrated that swine RNF5 interacted and ubiquitinated sABHD16A for degradation, which then attenuated the depalmitoylation effects on sIFITM1 and consequently promoted the activity of sIFITM1 against virus infection. Our findings provide new insights into the mechanisms of IFITMs fighting against virus; demonstrate the importance of RNF5, ABHD16A, and IFITM interplay in balancing antiviral immune responses; and provide broad-spectrum therapeutic strategies for animal viral diseases.

## MATERIALS AND METHODS

### Bioinformatics analysis

We utilized the AlphaFold2 algorithm through the ColabFold web server (accessed 31 March 2022 to 15 December 2022) (50) and the RoseTTAFold (51) algorithm through the Robetta web server (accessed 14 March 2022 to 15 December 2022) (52) to model the complete sequences of *Sus scrofa* RNF5 (A5A8Y3) and ABHD16A (A0A287AWB5). For subsequent analyses, we selected the top-ranked model that exceeded this threshold for each interaction prediction. We compared the top five outputs and selected Rank 1 to generate the figure. Structural figures were created using UCSF Chimera v1.17 (53).

We used Phyre2 (http://www.sbg.bio.ic.ac.uk/phyre2/) (29) and ubiquitination site prediction website GPS-Uber (http://gpsuber.biocuckoo.cn/) (38) to predict the possible transmembrane domain and ubiquitination site, respectively.

### Cell culture and transfection

Porcine kidney epithelial cells (PK15) and *Homo sapiens* embryonic kidney HEK293 cells were grown in high glucose (4.5 g/L) DMEM (#11995065; Gibco, USA) supplemented with 10% (vol/vol) fetal bovine serum, 100 U/mL penicillin, and 100 µg/mL streptomycin at 37°C in a humidified atmosphere containing 5% CO_2_. Transient transfections were performed with Lipofectamine 3000 (#L3000001; Invitrogen, USA) according to the manufacturer’s instruction using a 3:1 Lipofectamine-to-DNA ratio. For six-well plate scale experiments, approximately 5 × 10^5^ cells were seeded and a total of 1,250 ng of empty vector or recombination plasmids were introduced to each well after cells adhered to the plate for 12 h. In addition, Cell Counting Kit-8 (CCK8) assay was utilized for checking cell proliferation.

### Generation of sRNF5 and sABHD16A knockout cells

For generation of sRNF5 or sABHD16A knockout PK15 cells, guide RNA targeting exon 1 of swine RNF5 or ABHD16A was designed and synthesized (sRNF5: 5′-GAAGGGCCAAACCGCGAGCG-3′; sABHD16A: 5′-TCTACAAAATCTACCGGGAA-3′). Primers were annealed through 5-min boiling water bath and naturally cooled to room temperature treatment. Afterwards, the annealing primer was cloned into the LentiCRISPRv2-puro linearized vector through the BsmbI site.

PK15 cells were transfected with the recombination plasmid or control vector by using Lipofectamine 3000. Two micrograms per milliliters of puromycin dihydrochloride was added to screen transfected cells 24 h after transfection for 21 days. Monoclonal cells were obtained by the limited dilution culture method, and the knockout efficiency of sRNF5 or sABHD16A was checked via western blotting.

### Infection and antiviral assay

The JEV live vaccine strain SA14-14-2 (Wuhan Keqian Biology Company) and VSVΔG-GFP pseudotypes were propagated in Vero cells. The culture supernatants were harvested at 48 h post-transfection and filtered through 0.45-µm filters. Viruses at MOI of 0.1 were added to the medium of cultured cells 24 h post-transfection. After attachment for 1 h, the culture medium was removed, and cells were washed with phosphate buffered saline (PBS) twice and then immersed in DMEM medium containing 2% fetal bovine serum at 37°C for 24 h. Total cellular RNA was extracted, and the mRNA of the JEV and VSVΔG were measured by qRT-PCR using a pair of specific primers targeted at the JEV-E glycoprotein and VSV-nucleocapsid protein-coding sequence, respectively. Primers were listed below (5′-3′): JEV-E: forward: ACTGACATCTCGACGGTGGC; reverse: CTCCCAATCGCTTTACTGGT, VSV-N: forward: GATAGTACCGGAGGATTGACGACTA; reverse: TCAAACCATCCGAGCCATTC. Cellular proteins were extracted and subjected to western blotting with JEV-E glycoprotein (JE1) mouse monoclonal antibody and GFP mouse monoclonal antibody, respectively.

### Plaque formation assay

Briefly, transfected PK15 cells were incubated with the diluted JEV live vaccine strain SA-14-14-2 for 1 h at 37°C, the culture medium was removed, and cells were washed three times with PBS and immersed with complete DMEM containing 2% carboxymethyl cellulose sodium salt (#C488; Sigma-Aldrich) and 2% FBS. Five days later, cells were fixed with 4% paraformaldehyde fix solution and stained with crystal violet staining solution. Finally, the plaque sizes were calculated and analyzed via ImageJ.

### Plasmid construction

The cDNA of *rnf5*, *abhd16a*, and *ifitm1* from *Sus scrofa* (pig) were synthesized from the isolated total RNA of PK15 cells. To generate EGFP-, DsRed-, Flag-, Myc-, and HA-tagged sRNF5, sABHD16A, and sIFITM1, the cDNA fragments were digested with restriction endonuclease and individually cloned into the same sites of pEGFP-N1, pDsRed-monomer-N1, pcDNA3.1(+)-3×Flag, pcDNA3.1(+)-Myc, and pcDNA3.1(+)-HA vector, respectively. The expression plasmids of Flag-sRNF5(ΔTM), Flag-sRNF5(C42S), sABHD16A(K3R)-EGFP, sABHD16A(K452R)-EGFP, and sABHD16A(K3R; K452R)-EGFP were constructed by the overlap extension method (54). All plasmids were sequenced for verification.

### Quantitative real-time PCR gene expression analysis

The total RNA of PK15 cells was extracted with RNA-easy Isolation Reagent (#R701-01; Vazyme, China). One microgram of total RNA was reverse transcribed to cDNA by using the HiScript II Q RT SuperMix Kit (#R223-01; Vazyme) with oligo (dT) primers and a gDNA wiper to eliminate traces of genomic DNA. All real-time PCR reactions were carried out by using the StepOne Plus Real-Time PCR System (Applied Biosystems, USA) with a ChamQ SYBR qPCR Master Mix (#Q311-02; Vazyme). Relative expression was calculated by using a 2^−ΔΔCt^ method by normalizing to the expression of the swine *actb* housekeeping gene. Each experimental transcript was tested in triplicate. Samples without reverse transcriptase cDNA templates served as negative controls. Primers used were listed as follows: (5′-3′): *sabhd16A*: forward: TCCTCAACCAGGTCAAGAAGC; reverse: GCTGTCCCCCGCCGGTCCAC, *srnf5*: forward: TGGCAGCAGCGGAGGAGGAG; reverse: ACTGATGAAGACAGGGCCAAC.

### Confocal microscopy

Images were taken using a Leica TCS SP8 laser scanning confocal microscope with a Nikon Plan Apo ×60/1.5 oil lens objective. For ER staining, PK15 cells were rinsed with Hankʼs balanced salt solution with calcium and magnesium and then incubated with 1 µM ER Tracker Green (#E34251; Invitrogen). 0.5 h later, probes were removed and replaced with complete DMEM. To label lysosome, PK15 cells were incubated with 50 nM LysoTracker Red DND-99 (#L7528; Invitrogen) for 1 h and then washed with PBS twice and immersed in complete DMEM. To carry out the immunofluorescence experiments, cells were fixed with 4% paraformaldehyde (PFA) (for JEV-E antibody) or −20°C ethanol (for PSMD9 antibody) for 15 min, washed three times with PBS, and permeabilized with 0.1% Triton X-100 for 5 min. Cells were then blocked with 5% bovine serum album (BSA) in PBS. Both primary and secondary antibodies were applied onto cells and incubated for 2 h. After being washed three times with PBS, slices were mounted in an antifade mounting medium with Hoechst 33342 (#P0133; Beyotime, China). Finally, the imaging data were obtained with confocal microscopy.

### Bimolecular fluorescence complementation assay

Briefly, we cloned the cDNA fragment of sRNF5 and sABHD16A fused with YN (aa 1 to 155 of YFP) and YC (aa 156 to 239 of YFP), respectively (55). PK15 cells were transiently transfected with the following plasmids: YN + YC, YN + sABHD16A-YC, sRNF5-YN + YC, or sRNF5-YN + sABHD16A-YC. Twenty-four hours later, cells were stained with Hoechst 33342 for 10  min and subsequently examined using a confocal microscope. YFP signals denote the interaction between sABHD16A and sRNF5 in living cells.

### Imaging processing and analysis

Brightness and contrast were adjusted for images in ImageJ 1.54 f (National Institutes of Health, USA). A 1.0-pixel-wide median filter and a 50-pixel-wide rolling-ball background subtraction were used to increase the signal-to-noise ratio. Linear profile analysis was performed using a ImageJ plugin Plot Profile. To analyze the colocalization of two signals inside the cell, we used ImageJ Colocalization Finder and ScatterJ plugins (56) to analyze Pearson’s correlation coefficient.

### Reagents

The following inhibitors were used at a defined dose and time: the proteasome inhibitor MG-132 (#HY-13259; MedChemExpress, USA) at 10 µM for 12 h and the V-ATPase inhibitor Bafilomycin A1 (#S1413; Selleck Chemicals, USA) at 100 nM for 12 h.

### Western blotting

All cell lysates were prepared by washing cells twice with PBS and scraping them into RIPA lysis buffer (50 mM Tris pH 7.4, 150 mM NaCl, 1% Triton X-100, 1% sodium deoxycholate, and 0.1% SDS) supplemented with 1 mM PMSF, 10 mM DTT, 40 µg/mL DNase I, and 1 µg/mL of leupeptin, pepstatin, and aprotinin. Protein concentrations were measured with a BCA Protein Assay Kit, and equal amounts of total cell lysates were mixed with 6× SDS sample buffer, boiled, and subjected to SDS-PAGE. Proteins were transferred to a 0.45-µm PVDF membrane (#IPVH00010; Millipore, Germany) with a Trans-Blot Turbo transfer system (Bio-Rad, USA). The membrane was blocked in 5% BSA for 1 h at room temperature. Semi-quantitative analysis of the bands was carried out using an ImageJ gel analyzer. The mean gray value of band to internal reference band was considered as the relative protein expression. Three parallel wells were set for each protein sample.

The following antibodies were used in this study: RNF5 rabbit monoclonal antibody (dilution: 1:2,000; ab308066; Abcam), RNF5 mouse monoclonal antibody (dilution: 1:1,000; sc-81716; Santa Cruz), ABHD16A rabbit polyclonal antibody (dilution: 1:1,000; SRP08788; Saier Biotech), IFITM1 rabbit monoclonal antibody (dilution: 1:2,000; ab233545; Abcam), Japanese encephalitis virus E glycoprotein mouse monoclonal antibody (dilution: 1:2,000; ab41671; Abcam), PSMD9 mouse monoclonal antibody (dilution: 1:1,000; 67338-1-Ig; Proteintech), GFP mouse monoclonal antibody (dilution: 1:10,000; 66002-1-Ig; Proteintech), mCherry mouse monoclonal antibody (dilution: 1:5,000; 68088-1-Ig; Proteintech), Flag mouse monoclonal antibody (dilution: 1:5,000; F3165; Sigma-Aldrich), Myc rabbit polyclonal antibody (dilution: 1:1,000; 2272S; CST), HA rabbit polyclonal antibody (dilution: 1:5,000; 51064-2-AP; Proteintech), and GAPDH rabbit polyclonal antibody (dilution: 1:10,000; 10494-1-AP; Proteintech).

### Coimmunoprecipitation

Transfected PK15 and HEK293 cells were harvested and lysed with lysis buffer (20 mM Tris pH 7.4, 150 mM NaCl, 1% Triton X-100, 1% sodium deoxycholate, 1 mM PMSF, 10 mM DTT, 40 µg/mL DNase I, and 1 µg/mL leupeptin, pepstatin, and aprotinin). Cell lysates were performed by centrifugation at 10,000* g* for 10 min at 4°C, and the supernatant was collected. The Protein A+G magnetic beads (#P2108; Beyotime) binding with Flag M2 (for Flag-sRNF5 immunoprecipitation) were added to the reaction system and incubated at 4°C for 12 h. Afterwards, magnetic beads were collected by using a magnetic separation rack, then washed with TBS buffer (20 mM Tris, 137 mM NaCl, pH 7.6) three times, and boiled in SDS-PAGE loading buffer for 5 min. The supernatant was subjected to SDS-PAGE followed by western blotting.

To detect the interaction between endogenous sRNF5 and sABHD16A, normal mouse IgG (negative control) and mouse monoclonal RNF5 antibodies bound with Protein A+G magnetic beads were utilized to immunoprecipitate endogenous sRNF5 in PK15 cells.

### Ubiquitination detect assay

PK15 cells cotransfected with sABHD16A-EGFP and HA-sUB were lysed in lysis buffer containing 0.2% NP-40 followed by immunoprecipitation with GFP monoclonal antibody, after which the bead-bound proteins were washed and subjected to western blotting analysis with HA antibody.

### APEGS

The APEGS assay was performed using the method described by Percher et al. (32) and modified by our laboratory (6, 57). In brief, PK15 cells were transfected with expression plasmids for 24 h, and cells were collected and lysed with lysis buffer (50 mM triethanolamine , 150 mM NaCl, 4% SDS, 5 mM EDTA, and 1 µg/mL leupeptin, pepstatin, and aprotinin, pH 7.3). The cell lysate was incubated with 200 mM tris-(2-carboxyethyl) phosphine at 55°C for 1 h to reduce disulfide bonds. Afterwards, 1 M N-ethyl maleimide was added at 25°C for 4 h to block non-palmitoylated cysteines or free hydroxyl groups. All proteins were recovered twice by chloroform-methanol precipitation (methanol:chloroform: H_2_O = 4:1.5:3) and resuspended in lysis buffer. The mixtures described above continued to be incubated with 1 M NH_2_OH at 25°C for 4 h, which led to the cysteine sites being reduced to a free sulfhydryl state. The S-palmitate of cysteines was replaced by PEG-mal (5  kDa) at 25°C for 4 h. The S-palmitoylation blots of all test samples were detected with Flag (M2) monoclonal antibody through western blotting.

### Tet-on induction assay

To conduct the Tet-on induction experiment, we cloned the cDNA sequence of swine RNF5 into a pTight-mCherry-EF1-tetR-2A vector, which contains a multiple cloning site closely downstream of the Tet-responsive pTight promoter. The pTight promoter localized upstream of the minimal CMV promoter, which initiates target gene transcription with very low efficiency due to a lack of the enhancer. When Dox was added, rtTA was bound to Ptight, which activates the target gene expression (58).

For the induction of sRNF5-mCherry by the Tet-on system, PK15 cells were seeded on six-well plates at a density of 5*10^5^ cells per well and cultured overnight. Cells were then transfected with 1,250 ng pTight-sRNF5-mCherry-EF1-tetR-2A. Twenty-four hours later, 0.1/1/10 µg/mL Dox (#S4163; Selleck Chemicals) was added into the medium for 24 h, and the expression of sRNF5-mCherry was verified via SDS-PAGE.

### Protein half-life assay

The half-life of endogenous sABHD16A was determined by CHX chase assay. Fifty micrograms per milliliters of CHX (#M4879; Abmole, China) was added to the culture medium to inhibit protein synthesis. After 0, 4, 8, and 12 h, cells were harvested and the remaining sABHD16A protein was detected by western blotting.

### Statistical analysis

All data were presented as means ± SD. An unpaired two-tailed *t* test was used to determine significant differences between two groups, and one-way analysis of variance followed by Tukey’s *post hoc* test was used to evaluate differences between three or more groups. Data are mean ± SD from three independent experiments. Statistical significance was indicated as *P < 0.05, **P < 0.01, and ***P < 0.001; ns means not significant. All analyses were performed using GraphPad Prism 9.5 (GraphPad Software, USA).

## Data Availability

The data that support the findings of this study are available from the corresponding author upon reasonable request.
